# Preclinical Detection of Porcine Circovirus Type 2 Infection Using an Ultrasensitive Nanoparticle DNA Probe-Based PCR Assay

**DOI:** 10.1371/journal.pone.0097869

**Published:** 2014-05-19

**Authors:** Yong Huang, Xiujuan Zhang, Qian Du, Fengyu Wang, Xiaomin Zhao, Wenlong Zhang, Dewen Tong

**Affiliations:** College of Veterinary Medicine, Northwest A&F University, Yangling, Shaanxi, P. R. China; University of Missouri, United States of America

## Abstract

Porcine circovirus type 2 (PCV2) has emerged as one of the most important pathogens affecting swine production globally. Preclinical identification of PCV2 is very important for effective prophylaxis of PCV2-associated diseases. In this study, we developed an ultrasensitive nanoparticle DNA probe-based PCR assay (UNDP-PCR) for PCV2 detection. Magnetic microparticles coated with PCV2 specific DNA probes were used to enrich PCV2 DNA from samples, then gold nanoparticles coated with PCV2 specific oligonucleotides were added to form a sandwich nucleic acid-complex. After the complex was formed, the oligonucleotides were released and characterized by PCR. This assay exhibited about 500-fold more sensitive than conventional PCR, with a detection limit of 2 copies of purified PCV2 genomic DNA and 10 viral copies of PCV2 in serum. The assay has a wide detection range for all of PCV2 genotypes with reliable reproducibility. No cross-reactivity was observed from the samples of other related viruses including porcine circovirus type 1, porcine parvovirus, porcine pseudorabies virus, porcine reproductive and respiratory syndrome virus and classical swine fever virus. The positive detection rate of PCV2 specific UNDP-PCR in 40 preclinical field samples was 27.5%, which appeared greater than that by conventional and real-time PCR and appeared application potency in evaluation of the viral loads levels of preclinical infection samples. The UNDP-PCR assay reported here can reliably rule out false negative results from antibody-based assays, provide a nucleic acid extraction free, specific, ultrasensitive, economic and rapid diagnosis method for preclinical PCV2 infection in field, which may help prevent large-scale outbreaks.

## Introduction

Porcine circovirus type 2 (PCV2) is the major etiological agent of porcine circovirus associated diseases (PCVAD), including postweaning multisystemic wasting syndrome (PMWS), and porcine dermatitis and nephropathy syndrome (PDNS), porcine respiratory disease complex (PRDC), and congenital tremors type II (CT), which have caused heavy losses in global agriculture in recent years [1], [2], [3]. PCV2 serological studies showed that PCV2 infection is ubiquitous all over the world, while prevalence of clinical disease is relative lower, suggesting that subclinical or preclinical infection is the dominant form of PCV2 [4]. It has also been demonstrated experimentally that subclinical PCV2 infection may be associated with decreased vaccine efficacy [5]. Therefore, PCV2 subclinical infection not only is the most common infection form but also affect vaccine efficacy. Rapid and early identification of PCV2 subclinical infection is very important for the effective prophylaxis of PCVAD.

PCV2, belonging to the genus Circovirus of the family Circoviridae, are small nonenveloped DNA viruses containing a unique single-stranded circular genome of 1.7 kb [6]. The genomic DNA is packaged into a nonenveloped icosahedral capsid by capsid protein [7]. Antigenic studies have showed that PCV2 coat proteins possess six identified linear epitopes [8]. Sequence alignments of field isolated PCV2 capsid proteins have identified a number of variable regions corresponding to the identified epitope sites [9],[10],[11]. Indeed, these studies have demonstrated that antigenic differences in the capsid proteins exist among the different strains of PCV2 despite higher degree of sequence identity (90%) shared among their capsid proteins [12]. The antigenic difference exists among the different strains of PCV2 make it difficult to find an antibody that can be used to detect various PCV2 strains in field [13], but DNA probe targeted to the conserve sequence of different PCV2 strains can solve this problem. Therefore, to develop a DNA probe-based nanoparticle amplification method is very useful for detection of diverse PCV2 strains, especially for identification of PCV2 subclinical or preclinical infection.

In this study, we developed an ultrasensitive nanoparticle DNA probe-based PCR assay (UNDP-PCR) for preclinical identification of PCV2 infection via systematical optimization. Magnetic microparticles (MMP) coated with optimal specific PCV2 DNA probes and gold nanoparticles (AuNPs) coated with optimal specific PCV2 DNA probes and barcodes were used to enrich and amplify the weak signals from very small amount of PCV2 virus in serum samples. In each virus DNA-binding event, the gold nanoparticles carry with it a large number of DNA barcodes, and subsequently release these DNA barcodes to be detected by PCR. Therefore, the nanoparticle DNA probe-based PCR can significantly enhance the sensitivity of conventional PCR and to breakthrough the detection limit of conventional PCR, to gain an innovative method suitable for preclinical diagnosis of PCV2 infection, with greater sensitivity than the other conventional methods.

## Materials and Methods

### Materials, Reagents and Preclinical Samples

Carboxylated-modified magnetic beads (MyOne Dynabeads; Invitrogen, Inc., Carlsbad, CA, USA) were used for preconcentration of PCV2 DNA and separation of sandwich nucleic acid-complex. N-(3-Dimethylaminopropyl)-N′-ethylcarbodiimide hydrochloride and N-Hydroxysuccinimide were from Sigma-Aldrich (St. Louis, MO, USA) and used for activation of MyOne Dynabeads. DL-Dithiothreitol (DTT; Sigma-Aldrich, Inc., St. Louis, MO, USA) was used for the cleavage of oxidized thiolated oligonucleotides and release of thiolated barcode DNA from Au-NPs (Biopanda Reagents, Belfast, United Kingdom) surface.

A total of 40 blood samples were collected from the Landrace pigs (ranging from 1 to 2 months of age) without apparently clinical symptoms. These blood samples were provided by 2 local farms (xianyang & xingping) in the Shaanxi Province, China. The pigs were humanely euthanized with a dose of Ketamine (15 mg/kg. i.v. in the jugular venous external), then 5–10 ml of blood samples from each pig were collected by jugular venipuncture. The serum samples were tested using the commercial ELISA, PCR, real-time PCR and UNDP-PCR. 40 peripheral blood samples were identified as PCV2-negative by commercial PCV2 ELISA kit (Lvshiyuan Biotechnology Co., China). 2 samples were identified as positive by conventional PCR and sequencing and the other 9 samples were further identified by PCR after virus isolation, infection, culture and amplification [14].

This experiment was carried out according to the Animal Ethics Procedures and Guidelines of the People’s Republic of China. These works was approved by the Ethical Committee for Animal Experiments of the Northwest A&F University. No other specific permissions were required for these activities. This study did not involve endangered or protected species.

### Virus Strains and Propagation

Different serotypes and genotypes of PCV2 strains (Table 1) used in this study were isolated, purified and stocked in our lab [14], [15]. Porcine parvovirus (PPV) YL strain and porcine reproductive and respiratory syndrome virus (PRRSV) Shaanxi strain used in this study were isolated previously by our team [16], [17] Vaccine strain of porcine pseudorabies virus (PRV) (Cat. no.160027018) were purchased from Luoyang Pu-Like Bio-Engineering Co., and propagated in the porcine kidney cell line (PK-15). The Shimen Strain of classical swine fever virus (CSFV) was provided kindly by Professor yanming Zhang [18]. Porcine circovirus type 1 (PCV1) were isolated from a PK-15 cell line infected persistently with PCV1. These viruses were used as standard viruses for the UNDP-PCR assay and maintained at −80°C until using. PCV2, PPV, CSFV and PRV were propagated in the PCVl-free PK-15 cells, PRRSV was propagated in the Marc-145 cells. These virus were identified by conventional PCR or RT-PCR specifically for these virus and sequencing following previous study [14], [19].

**Table 1:** Different serotypes and genotypes of porcine circovirus type 2 used in this study.

PCV2 strains	Serotype	Genotype	Orgin
AF381176		2a	China
DQ104423		2a	China
AF112862		2a	Canada
EU366323	1A/1B	2b	China
AY579893	1A/1B	2b	China
AY391729	1A/1B	2b	China
FJ644927	1C	2b	China
AY291317	1C	2b	China
AY484410	1C	2b	UK
AY181947	1C	2b	China
KC800634		Recombinant	China
KC800636		Recombinant	China
KC800639		Recombinant	China
KC800644		Recombinant	China
KC800646		Recombinant	China

### Extraction of DNA and RNA

Viral genomic DNA and RNA were extracted from cell cultures infected with each virus or serum samples using E.Z.N.A. HP Viral RNA/DNA Kit (OMEGA, USA) according to the manufacture’s instruction. After extraction, DNA/RNA was eluted in 50 µl of elution buffer and stored at −20°C. Complementary DNA (cDNA) was synthesized using 2 µg of the eluted RNA with random primers and the reverse transcriptase kit (Takara Corp., Japan) according to the manufacturer’s instructions.

### Primers and Probes in this Study

Primers for conventional PCR detection of PCV2, PCV1, PPV, PRRSV, CSFV and PRV were present in Table 2. Primers and probes for UNDP-PCR detection system were designed by comparing multiple sequences of PCV2a- and PCV2b-encoding gene using DNASTAR software package (DNASTAR, inc., Wisconsin, USA). The highly conserved sequences in the capsid protein-coding region of different PCV2a and PCV2b strains were selected for designing primers and probes (Table 2). The designed primers and probes have higher specificity to ensure precise diagnosis. The primers and probes were synthesized by sangon (Shanghai, China).

**Table 2:** Primers and probes used in this study.

Label	Orientation	Assay	Sequence (5′–3′)	Position
PEGFP-F	Forward	LINK-PCR	ATAGCGGTTTGACTCACGGG	400–419
PEGFP-R	Reverse	LINK-PCR	CCCTTTGACGTTGGAGTCCA	1839–1820
PCV2DP-1	Forward	Detect PCR	AGCAGAAGAACGGCATCAAG	
PCV2DP-2	Reverse	Detect PCR	GCCATCTTGGCCAGATCCT	
PCV2F	Forward	Conventional-PCR	CCTTTGTTACAAAGTTAT	1338–1355
PCV2R	Reverse	Conventional-PCR	TTGGCCAGATCCTCCGCCGCCGC	1657–1679
PCV1F	Forward	Conventional-PCR	GGATTTGAAGCAGTGGA	929–945
PCV1R	Reverse	Conventional-PCR	GCGGAGAAGACCATATT	1655–1639
PPVF	Forward	Conventional-PCR	AGTTAGAATAGGATGCGAGGAA	1761–1782
PPVR	Reverse	Conventional-PCR	AGAGTCTGTTGGTGTATTTATTGG	2026–2002
PRRSVF	Forward	Conventional-PCR	GAGTTTCAGCGGAACAATGG	14359–14379
PRRSVR	Reverse	Conventional-PCR	GCCGTTGACCGTAGTGGAG	14810–14791
CSFVF	Forward	Conventional-PCR	GTCGTCAGTAGTTCGACG	182–200
CSFVR	Reverse	Conventional-PCR	ATGCTCTTTTGGGGCTAT	959–941
PRVF	Forward	Conventional-PCR	GGGGTTGGACAGGAAGGACACCA	17205–17228
PRVR	Reverse	Conventional-PCR	AACCAGCTGCACGCGCTCAA	17403–17383
Probe 1		Hybridization	5′NH_2_-T_15_ ATCTTAAAGACCCCCCACTTAA	1040–1061
Probe 2		Hybridization	5′NH_2_-T_15_ ATACGACCAGGAATACAATATCCGTGTAACC	1087–1117
Probe 3		Hybridization	5′NH_2_-T_15_ GTAGGCCTCGGCACTGCGTTCG	1128–1149
Probe 4		Hybridization	5′NH_2_-T_15_ CTGTTATTCTAGATGATAACTTTGTAACAAAGG	1338–1370
Probe 5		Hybridization	5′NH2-T_15_ AAGGTTAAGGTTGAATTCTGGCCCTGCTC	1409–1437
Probe 6		Hybridization	5′NH_2_-T_15_ ACCCGCCTCTCCCGCACCTTCGGATATACT	1567–1596
Probe 7		Hybridization	5′NH_2_-T_15_ CCACCGTTACCGCTGGAGAAGGAAAAATGGC	1606–1636
Probe 8		Hybridization	5′NH_2_-T_15_ CCAGATCCTCCGCCGCCGCCCCTGGCTCGTC	1645–1675
Probe 9		Hybridization	5′NH2-T_15_GATCCTCCGCCGCCGCCCCTGGCTC	1648–1672
Probe 9		Hybridization	5′SH- T_15_GATCCTCCGCCGCCGCCCCTGGCTC	1648–1672
Barcode		Amplification	TTACTTGTACAGCTCGTCCATGCCGAGAGTGATCCCGGCGGCGGT	694–738
Oligo1		Hybridization/Amplification	5′SH-T_15_ GATCCTCCGCCGCCGCCCCTGGCTC TTACTTGTACAGC TCGTCCATGCCGAGAGTGATCCCGGCGGCGGT	
Oligo2		Hybridization/Amplification	5′SH-GATCCTCCGCCGCCGCCCCTGGCTC TTACTTGTACAGCT CGTCCATGCCGAGAGTGATCCCGGCGGCGGT	
Oligo3		Hybridization/Amplification	5′SH- T_60_ GATCCTCCGCCGCCGCCCCTGGCTC TTACTTGTACAG CTCGTCCATGCCGAGAGTGATCCCGGCGGCGGT	
Oligo4		Hybridization/Amplification	5′SH-T15 GATCCTCCGCCGCCGCCCCTGGCTCTTACTTGTACAGCTCG	

### Preparation of PCV2 Specific Probe-coated Magnetic Microparticles (MMP) for UNDP-PCR Assay

The optimal probes for capture of PCV2 genomic DNA were determined by testing different serotypes and genotypes of PCV2 strains (Table 1). The 5′ amino (NH2)-modified PCV2 specific oligonucleotide probes were coupled covalently to the carboxylated-modified MyOne Dynabeads by using water-soluble N-Ethyl-N′-[3-dimethylaminopropyl] carbodiimide hydrochloride (EDC) to establish an amide bond. The detailed methods were according to the manufacturer’s protocol [20]. Functionalized Dynabeads were resuspended in TE buffer to 10 mg/mL and were stored at 4°C until use. Functionalized Dynabeads prepared in this manner retained their activity over 5 months.

### Preparation of PCV2 Specific Probe Barcode-coated Gold Nanoparticles (AuNPs)

The 5′ sulfydryl (SH)-modified PCV2 specific oligonucleotide probes/barcodes were mixed with thiolated AuNPs to form a strong covalent Au-S bond spontaneously between the two components. Briefly, 1 ml of 15 nm-diameter AuNPs (10 nmol/L) were centrifuged at 15,700 g for 30 min, and then resuspended in 100 µL ddH_2_O. Then the particles were modified subsequently with thiolated DNA (final concentration, 3 µmol/L) by slow salt aging (48 h) to a final concentration of PBS (0.1 M NaCl in 0.01 M of phosphate buffer, pH 7; denoted as PBS unless indicated otherwise). Unbound thiolated DNA was removed by repetitive centrifugation (15,000 g) and rinsing of the particles. The functionalized AuNPs were resuspended in 0.01 M PBS and were stored at 4°C until use.

### Detection Procedure of UNDP-PCR Assay

The serum samples containing PCV2 were added same volume PBS and boiled for 10 min to release viral DNA. The DNA sample were add in a hybridization tube which containing 2 µL of probe-coated MMP and 5× hybridization buffer (5×SSC, 0.1% Tween-20 and 2% SDS in H_2_O), followed by vortex, and incubation at 40°C for 30 min with vigorous stirring. Then 2 µL probe/barcode-coated AuNPs were added and incubated at 50°C for 30 min with stirring. Tubes were then transferred to magnetic wells for 5 min. The AuNP-MMP complexes were magnetically separated and washed two times with 1 mL of 1×hybridization buffer and two times with 1 mL of PBS to remove the hybridization buffer and unbound probe/barcode-coated AuNPs. The sandwich complexes containing the barcode DNA were then detected by PCR.

The oligonucleotides in the AuNP-MMP complexes were eluted from the gold nanoparticle surface with 100 µL of elution buffer (0.5 mol/L DTT, 10 mM Tris-HCl, 1 mM EDTA, pH 7.5) at room temperature for 10 min, Then the eluted oligonucleotides were purified using absolute alcohol and subject to quantification by conventional and real-time PCR. Conventional PCR was performed in a final volume of 50 µl containing Premix Ex TaqTM (TaKaRa, Dalian, China), 0.4 µM forward primer, 0.4 µM reverse primer, 2 ng of specific capture DNA templates and the eluted oligonucleotides under the following conditions: 95°C for 5 min followed by 35 cycles of 95°C for 20 s, 55°C for 20 s and 72°C for 40 s followed by a final incubation at 72°C for 5 min. Following PCR, 25 µL of amplified product was separated on a 1% agarose gel. Respective gels were stained with ethidium bromide and the PCR products visualized under UV light. For different barcodes identification by real-time PCR, amplification was performed as described previously [21]. Primers Bar1-F (5′-TTACTTGTACAGCTCGTC-3′) and Bar1-R (5′-ACCGCCGCCG GGATCACT-3′) and probe Bar1-p (FAM-5′-CTCGGCAT-3′-TAMRA), primers Bar2-F (5′-TTGATCCTCCGCCGCCGC-3′) and Bar2-R (5′-CGAGCTGTACAAGTAA-3′) and probe Bar2-p (FAM-5′-GAGCCAGG-3′-TAMRA). Fluorescence data were recorded in real-time with an IQ5 multicolor real-time PCR detection system (Bio-Rad, USA).

### The Sensitivity, Specificity, and Reproducibility of the UNDP-PCR Assay

PCV2 complete genome sequence was amplified from the genomic DNA of PCV2 strain (GenBank No. EU366323) using the forward primer (5′-GAACCGCGGGCTGGCTG AACTTTTGAA-3′) and reverse primer (5′-GCACCGCGGAAATTTCTGACAAACGTTAC A-3′). The PCR products were purified and cloned into pGEM-T Easy Vector (Promega, USA) to construct pGEM-PCV2, and then sequenced by sangon (Shanghai, China). The concentrations of plasmid were determined by Nanodrop 2000 Spectrophotometer (Thermo scientific, USA). Copy number was calculated using the following formula:amount (copies/µL) = 6×10^23^ (copies/mol) × concentration (g/µL)/MW (g/mol). To test the sensitivity, pGEM-PCV2 plasmid and PCV2 serum were serially diluted from 1 to 10^10^ copies/µL and 1 to 10^5^ copies/ml, respectively, and then tested by conventional PCR and UNDP-PCR. To test the specificity, PRRSV, PRV, PPV, CSFV, and PCV1 were tested by UNDP-PCR. To test the reproducibility of this assay, serial diluted PCV2 (GenBank No. EU366323) samples prepared in serum were tested by the inter- and intra-assay. The inter-assay and intra-assay were performed in three replicates of each run by three independent tests in consecutive three days.

## Results

### Assay Design and Optimization

**Figure 1:**
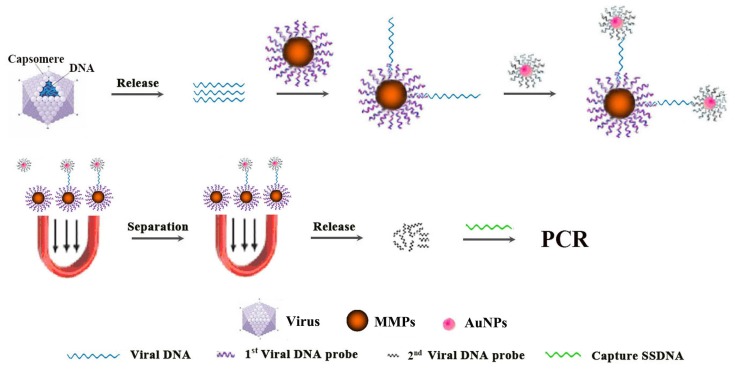
Schematic of the ultrasensitive nanoparticle DNA probe-based PCR (UNDP-PCR) assay.

To find an ultrasensitive, economic and rapid diagnosis method for preclinical PCV2 infection in field, a set of experiments was carried out to establish a protocol for the assay schematically depicted in Fig. 1.

**Figure 2:**
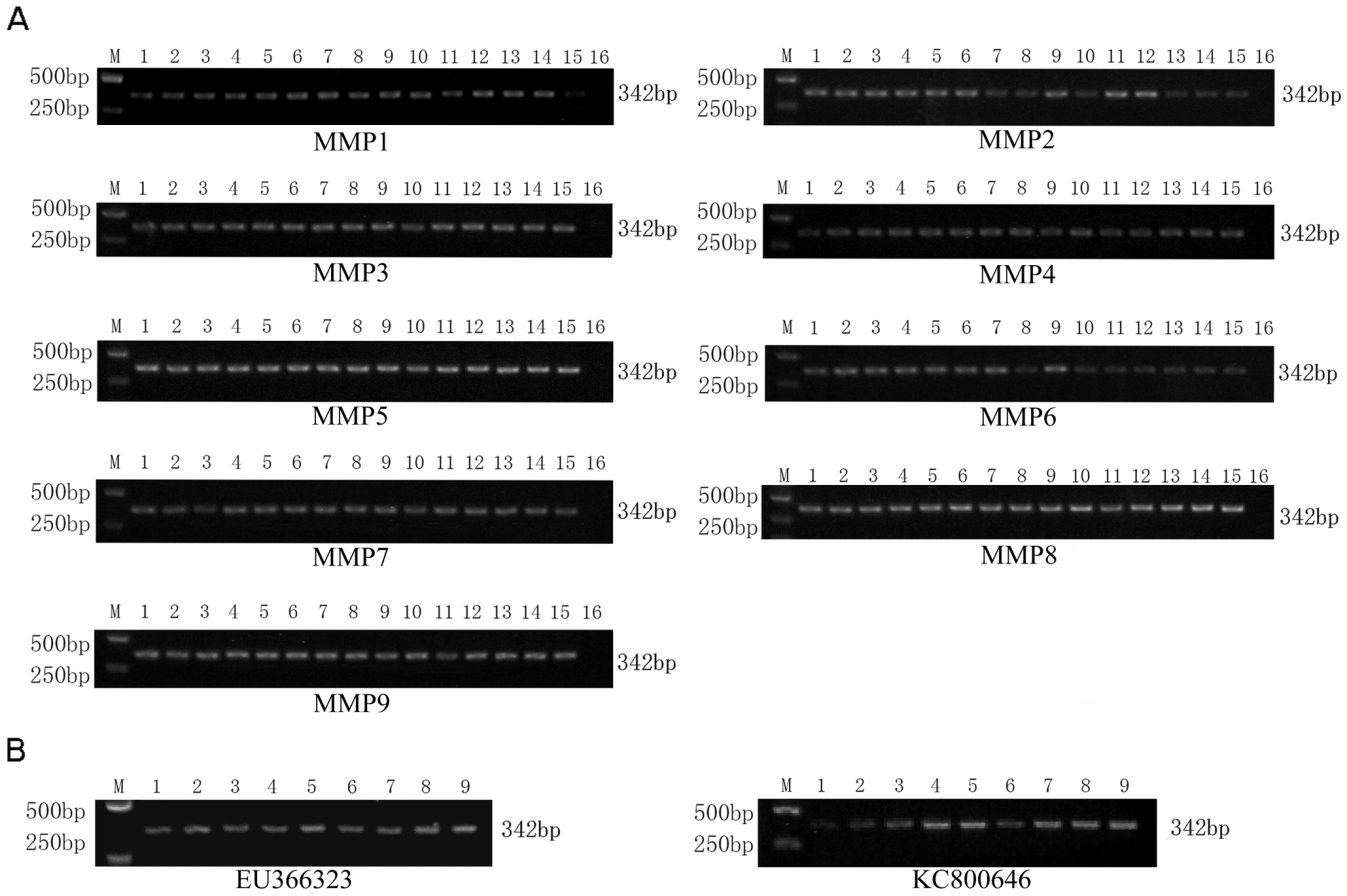
Comparison of different hybridization probes functionalized magnetic microparticles. (**A**) Functionalized magnetic microparticles MMP-p1, -p2, -p3, -p4, -p5, -p6, -p7, -p8 and -p9 were incubated with the DNA derived from different PCV2 representative strains in hybridization buffer at 40°C for 30 min, followed by washing and magnetic separation. MMP-DNA complex were detected by PCV2-specific PCR and PCV1-specific PCR. M: Trans 2000 Plus DNA Marker; 1: AF381176; 2: DQ104423; 3: AF112862; 4: EU366323; 5: AY579893; 6: AY391729; 7: FJ644927; 8: AY291317; 9: AY484410; 10: AY181947; 11: KC800634; 12: KC800636; 13: KC800639; 14: KC800644; 15: KC800646; 16: AY193712 (PCV1). (B) Identification of different hybridization probes functionalized MMPs. The indicated two PCV2 representative strains were incubated with MMP-p1, -p2, -p3, -p4, -p5, -p6, -p7, -p8 and -p9, respectively, followed by PCV2 specific PCR detection. M: Trans 2000 Plus DNA Marker; 1: MMP1; 2: MMP2; 3: MMP3; 4: MMP4; 5: MMP5; 6: MMP6; 7: MMP7; 8: MMP8; 9: MMP9.

Firstly, to expand the breadth of UNDP-PCR assay as much as possible, we selected the highly conserved sequence of length 18–35 bp in the capsid protein-coding region from about 100 different PCV2a, PCV2b and recombination strains, and aligned with PCV1 to exclude the regions with higher homology with PCV1. At last, we determined 8 targeted regions for designing probes 1–8 (Table 2). The designed probes 1–8 were coated to MMP, respectively, to prepare functionalized magnetic microparticles MMP-p1, -p2, -p3, -p4, -p5, -p6, -p7 and -p8. The optimal probes for capture of PCV2 genomic DNA were selected and determined by testing the capture efficiency of these MMPs for genomic DNAs from different serotypes and genotypes of PCV2 strains. All of eight MMP-ps could capture 15 representative PCV2 strains derived from different genotypes and serotypes (Table 1), but did not show cross reaction with PCV1, suggesting these eight probes have higher specificity to PCV2 (Fig. 2A). In these probes-functionalized MMPs, however, MMP-p1, -p3 and -p4 showed relatively lower capture ability for most of representative PCV2 strains DNA, MMP-p2 showed lower capture ability for PCV2b-1C strains (FJ644927, AY291317, AY181947) and PCV2 recombinants (KC800639, KC800644, KC800646), MMP-p6 showed lower capture ability for PCV2a strain (AF381176), PCV2b-1C strain (AY291317, AY181947) and PCV2 recombinants (KC800634, KC800636, KC800639, KC800644, KC800646), MMP-p7 showed lower capture ability for PCV2a strains (AF112862), PCV2b-1C strain (AY181947) and PCV2 recombinant (KC800646), whereas MMP-p5, -p8 and -p9 showed higher capture ability for most of representative PCV2 strains DNA (Fig. 2A, B). Moreover, MMP-p9, containing a shorter p8, also showed higher capture ability for most of representative PCV2 strains DNA. Thus, probe 5-functionalized magnetic microparticles are optimal for preconcentration of various PCV2 strains DNA and separation of sandwich nucleic acid-complex in this assay.

**Figure 3:**
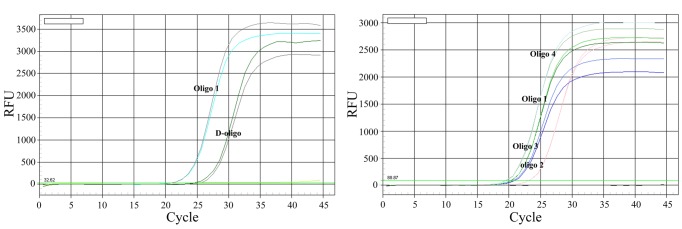
Comparison of different oligos functionalized Au-NPs. The genomic DNA of PCV2 strain (GenBank No. EU366323) were incubated with MMP5 at 40°C for 30 min with vigorous stirring. Then D-oligo (probe9+Barcode), oligo1, oligo2, oligo3 and oligo4 functionalized Au-NPs were added and incubated at 50°C for 30 min with stirring. After magnetic separation, barcode DNA and oligo1 were then detected by real-time PCR using bar prime1 and bar probe1 (left panel), while oligo1–4 were then detected by real-time PCR using bar prime2 and bar probe2 (right panel).

Next, we evaluated the conjugation and amplification efficiency of functionalized Au-NPs with different oligonucleotides in UNDP-PCR assay. Au-NPs coated with both SH-modified probe 9 and barcode 1 (double oligos, D-oligo) showed a relative lower amplification efficiency via quantification of barcode DNA by real-time PCR when compared with the Au-NPs coated with oligo 1 (synthezied probe 9 and barcode 1 in one oligo) (Fig. 3 left panel). Oligo 2- and oligo 3-functionalized Au-NPs also appeared a reduced amplification efficiency compared with the Au-NPs coated with oligo 1, whereas oligo 4-functionalized Au-NPs showed higher amplification efficiency than oligo 1-functionalized Au-NPs (Fig. 3 right panel), suggesting that oligo 4-functionalized Au-NPs is optimal for binding and amplification of detected DNA signals in this assay.

### The Detection Breadth of UNDP-PCR

**Figure 4:**
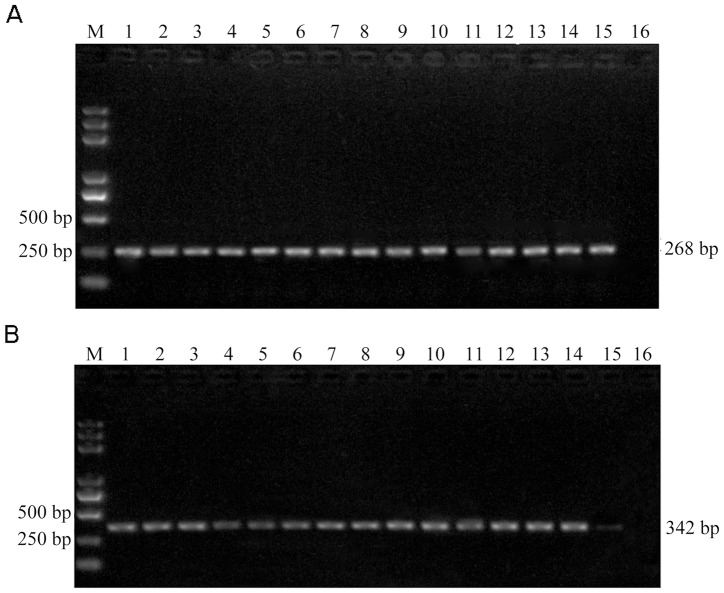
Analysis of the detection breadth of UNDP-PCR. (A) Functionalized MMP-p5 were incubated with the DNA derived from different PCV2 representative strains in hybridization buffer at 40°C for 30 min, then oligo4 functionalized Au-NPs were added and incubated at 50°C for 30 min with stirring. After washing and magnetic separation, oligo4 were then detected by PCR as described in Methods. (B) PCV2 poly antibodies functionalized magnetic microparticles incubated with different PCV2 representative strains in assay buffer (1% BSA, 1X PBS, 0.2% Tween20 and 2% sheep serum). Antigen-antibody immune complexes were separated magnetically and were washed twice with 1 ml of magnetic-probe solution that contained PBS pH 7.4, 0.1% BSA and 0.05% Tween20. Then viral DNA were extracted by Viral DNA purification Kit, followed by PCV2-specific PCR detection. Lane: M: Trans 2000 Plus DNA Marker; 1: AF381176; 2: DQ104423; 3: AF112862; 4: EU366323; 5: AY579893; 6: AY391729; 7: FJ644927; 8: AY291317; 9: AY484410; 10: AY181947; 11: KC800634; 12: KC800636; 13: KC800639; 14: KC800644; 15: KC800646; 16: AY193712 (PCV1).

The breadth of UNDP-PCR were determined by measuring the presence of PCV2 genomic DNA in 15 diverse PCV2 strains, including 3 strains from PCV2a, 7 strains from PCV2b and 5 strains from emergent PCV2 recombinants. Accordingly, all of DNAs from 15 strains are perfectly captured and detected by this method (Fig. 4A). As a control, however, using PCV2 antibodies functionalized MMPs did not perfectly capture individual PCV2 strains, such as PCV2 recombinant KC800646 (Fig. 4B). These results suggest that DNA probe-based UNDP-PCR methods posses more wide detection breadth compared with antibody-based nanoparticle-amplification approaches in detection of some DNA virus.

### The Specificity of UNDP-PCR

**Figure 5:**
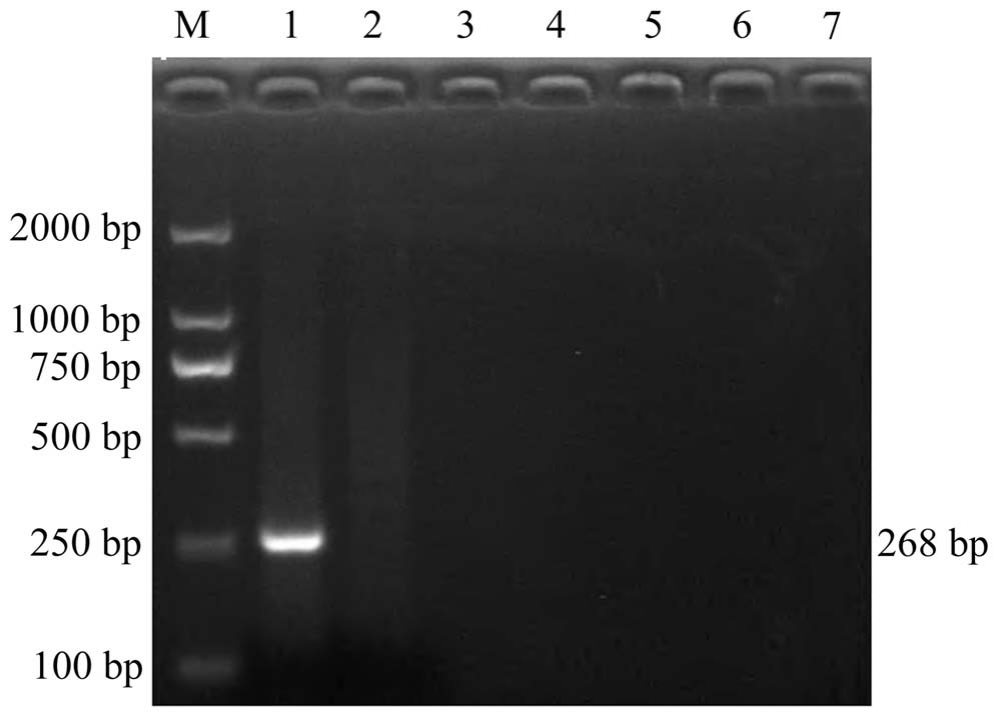
Analysis of the specificity of UNDP-PCR for detection of PCV2. PRRSV, PRV, PPV, CSFV, PCV1 and the blood DNA of healthy swine were tested by UNDP-PCR as control. Lane M: DL2000 DNA Marker; lane 1: PCV2; lane 2: the blood DNA of healthy swine; lane 3: PCV1; lane 4: PPV; lane 5: PRV; lane 6: PRRSV; lane 7: CSFV.

The genomic nucleic acids of the PCV2, PCV1, PPV, PRV, CSFV and PRRSV, and blood DNA of healthy animals were extracted and used to assess the specificity of UNDP-PCR assay for PCV2. Agarose gel electrophoresis analysis showed that the PCV2 specific UNDP-PCR assay only appeared a positive reaction with PCV2 DNA, and did not detect the presence of the DNA of PCV1, PPV, and PRV, and the cDNA of PRRSV and CSFV, and showed a negative reaction with the blood DNA of healthy swine (Fig. 5). These results suggest that UNDP-PCR assay for PCV2 is only specific for PCV2 infection without cross-reactivity with porcine circovirus type 1, porcine parvovirus, porcine pseudorabies virus, porcine reproductive and respiratory syndrome virus and classical swine fever virus.

### The Sensitivity and Reproducibility of UNDP-PCR

**Figure 6:**
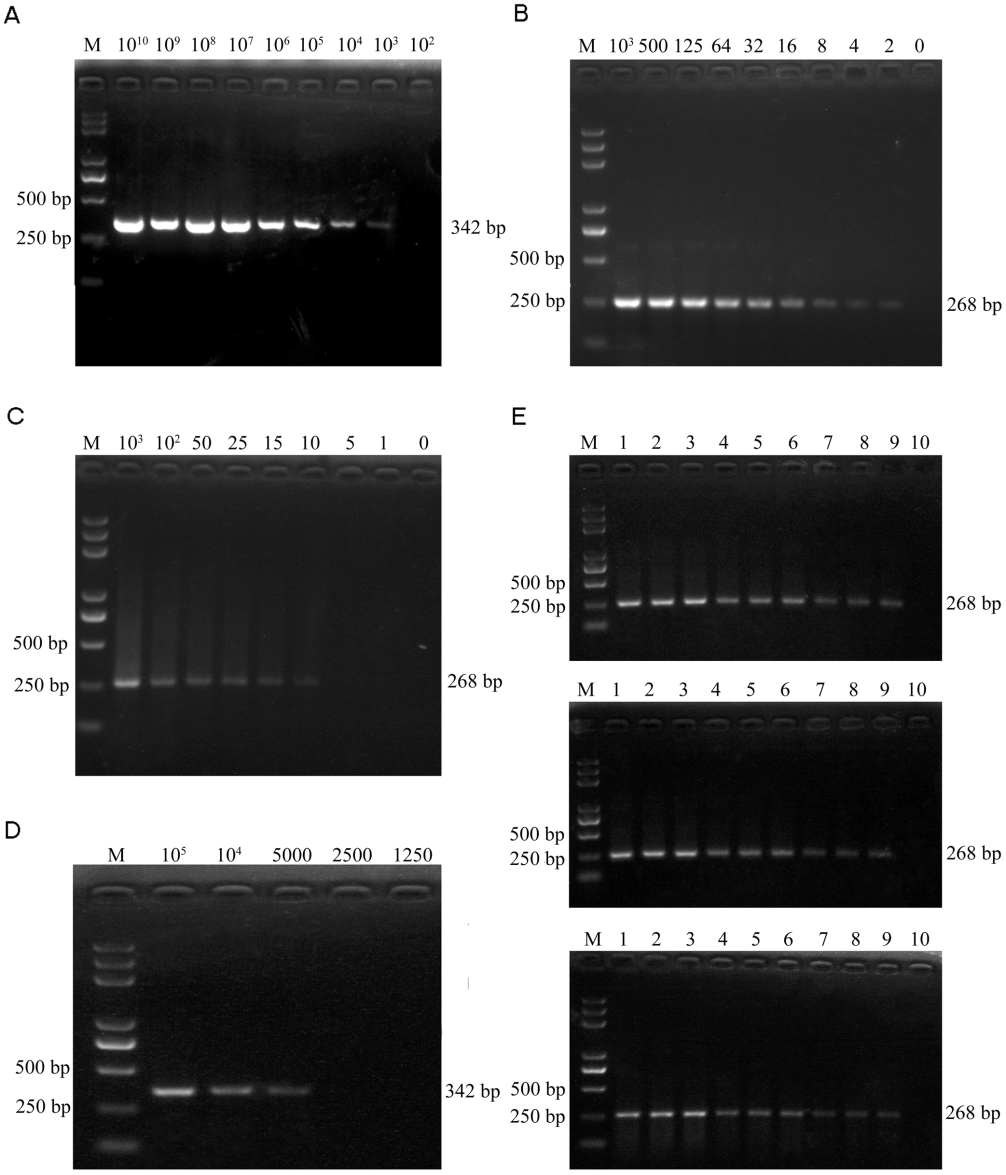
Analysis of the sensitivity and reproducibility of UNDP-PCR. (A) Serial dilutions of PCV2 plasmid DNA were detected by conventional PCR. (B) Serial dilutions of PCV2 plasmid DNA were detected by UNDP-PCR. (C) Serial dilutions of PCV2 serum samples were detected by UNDP-PCR. (D) Serial dilutions of PCV2 serum samples were detected by conventional PCR. (E) Indicated three concentration of PCV2 serum samples were detected by UNDP-PCR in triplicates and in three independent runs. Agarose gel electrophoresis data in upper, middle and lower panels are representative three independent experiments. Lane M: Trans 2000 Plus DNA Marker; lane 1–3∶5×10^3^; lane 4–6∶5×10^2^; lane 7–9∶5×10^1^; lane10: negative samples.

A Series of 10-fold dilutions of PCV2 plasmid DNA were tested firstly. The detection limits for conventional PCR were 10^3^ copies of plasmids (Fig. 6A), while 2 copies of plasmids could be detected by UNDP-PCR (Fig. 6B). Furthermore, serial dilutions of purified PCV2 in serum were used to assess the sensitivity of UNDP-PCR assay. Viral DNA were released from the serum containing PCV2 by heat lysis and then detected by UNDP-PCR. The specific 268 bp PCR amplicon was separated by agarose electrophoresis and quantified by the computer assisted image analyzer. As shown in Fig. 6C, lane 2 to 9 represent amplicons of the samples with PCV2 concentrations ranged from 10^3^ copies/ml to 1 copy/ml, and lane 10 is the negative control serum without PCV2. As our prediction, 10 copies/ml of PCV2 sample could be detected by this assay, whereas over 5×10^3^ copies/ml of PCV2 serum sample could be detected by conventional PCR (Fig. 6D).

The reproducibility of the UNDP-PCR assay was evaluated by testing 3 different concentrations of PCV2 serum samples in triplicates and in three independent runs for consecutive three days. 3 independent replicates assays of the UNDP-PCR showed highly consistent results (Fig. 6E).

### Application of UNDP-PCR in Pre-clinical Detection

**Figure 7:**
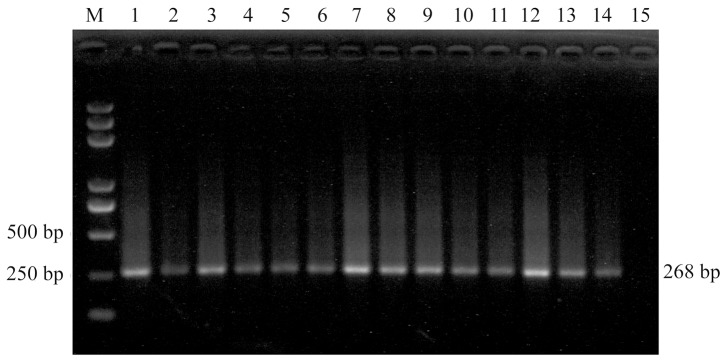
Agarose gel electrophoresis of the relative viral load levels of 11 positive preclinical specimens identified by UNDP-PCR. Lane M: Trans 2000 Plus DNA Marker; lane 1–11: preclinical specimens; lane 12∶5×10^3^ Standards; lane 13∶5×10^2^ Standards; lane 14∶5×10^1^ Standards; lane15: negative samples.

To evaluate the ability of pre-clinical detection for PCV2 by UNDP-PCR, 40 preclinical serum samples collected from epidemic farms were detected by PCR, real-time PCR and UNDP-PCR assay. Among 40 samples, 11 samples were found to be positive by UNDP-PCR, two samples were positive by conventional PCR and five samples were positive by real-time PCR (Table 3). The 11 positive samples detected by the UNDP-PCR method included all of the samples found to be positive by the other methods (i.e., conventional and real-time PCR). The detection rate of conventional PCR, real-time PCR, and UNDP-PCR were 5%, 12.5%, and 27.5%, respectively. Furthermore, the levels of PCV2 viral loads in different positive samples was determined by comparing the relative band intensity of the unknown to the standard band intensity obtained by adding serial diluted PCV2 to serum (range: 50–5000 copies/ml of serum). Results showed that among 11 positive samples, the viral loads of two samples were over 5000 copies/ml, three samples were between 5000 and 500 copies/ml, five samples were between 500 and 50 copies/ml, and one sample was below 50 copies/ml (Fig. 7).

**Table 3:** Comparison of the detection rate of PCV2 infected preclinical samples by conventional PCR, real-time PCR and UNDP-PCR methods.

	Conventional PCR	SYBR Green real-time PCR	UNDP-PCR
Number of positive samples	2	5	11
Number of negative samples	38	35	29
Positive rate (%)	5	12.5	27.5

Taken together, these results suggest the PCV2 specific UNDP-PCR assay developed here might be the most sensitive, economic and rapid method for preclinical diagnosis of PCV2 infection and evaluation of infection levels.

## Discussion

Technological advances in nanotechnology provide the opportunity to overcome many shortcomings of other diagnostic methods [22]. The ultrasensitive nanoparticle DNA probe-based PCR assay (UNDP-PCR) provides an innovative approach to detect target virus that overcomes many shortcomings of other diagnostic approaches. It has more wide detection breadth compared with antibody-based nanoparticle-amplification approaches, can detect 15 representative PCV2 strains of different genotype and serotype. It has higher sensitivity and selectivity compared with other PCR-based approaches and without the need for nucleic acid extraction.

This method offers several advantages over existing PCV2 or other DNA viruses detection methods [23], [24], [25]. First, unlike the conventional PCR, real-time PCR and Loop-mediated isothermal amplification (LAMP), the target-binding reaction portion of the UNDP-PCR assay can be performed in alternative reaction volume from 10 µl to 10 ml, even more (as opposed to the biggest volume of 50 µl or 100 µl in a PCR, real-time PCR or LAMP reaction). Accordingly, a large quantity of specific probes-functionalized magnetic microparticles and gold nanoparticles can be added to facilitate binding kinetics between the specific probes and target virus DNA, subsequently, the functionalized gold nanoparticles carry with it a large number of DNA probes and barcodes per viral DNA-binding event, which provide several thousands of barcode DNA as templates for subsequent PCR detection. Therefore, there is substantial amplification and virus DNA can be detected below attomolar concentration. Second, sample pre-treatment and enrichment is crucial for detection of biosamples with an extremely low concentration. In conventional PCR, real-time PCR and LAMP assays, the DNA of virus samples is usually extracted utilizing viral DNA purification kits, which are labor-intensive and expensive procedures, particularly in the detection of large number of samples. However, the developed UNDP-PCR system can directly capture and enrich targeted viral DNA from the lysate of serum samples by using specific probes-functionalized magnetic beads without the needs to extract viral DNA. Third, the DNA probe based nanoparticle-amplification method is exquisitely specific because DNA probes bound to the gold nanoparticles and the magnetic microparticles are directed against two distinct targeted virus genomic DNA sequence. Forth, unlike the conventional ELISA assay, the target-binding reaction of the UNDP-PCR assay is performed in solution (as opposed to on the flat surface of an ELISA plate). Thus a large volume of serum samples can be detected in each reaction, which makes this method faster and more efficient than ELISA. In addition, the detection sensitivity and breadth of ELISA are limited. Lastly, because the method is very sensitive and DNA extraction free, short incubation times are sufficient for the DNA-probes binding as found in other nanoparticle-based PCR assays [26]. Thus, the whole assay could be completed within 6 h using pre-functionalized nanoparticles.

In this proof-of-concept study, the nanoparticle DNA probe-based PCR assay could detect 2 copies of PCV2 recombinant plasmid per reaction and 10 viral copies of PCV2 in per ml serum, whereas conventional PCR just could detect over 1×10^3^ copies of PCV2 recombinant plasmid per reaction and 5×10^3^ viral copies of PCV2 in per ml serum. Real-time PCR had been reported to have a linear detection range of 34.1 to 10^8^ copies of PCV2 recombinant plasmid per reaction [24] and a linear detection range of 2.2×10^3^ to 2.2×10^10^ copies of PCV2 per ml serum [23]. In pre-clinical application, UNDP-PCR detected PCV2 in serum from 11 out of 40 preclinical infection subjects, only 2 out of 11 PCV2 positive subjects with more than 5000 copies of PCV2 in per ml serum were detected by conventional PCR, 5 out of 11 PCV2 positive subjects with more than 500 copies of PCV2 in per ml serum were detected by real-time PCR, whereas the subjects with less than 500 copies of PCV2 in per ml serum (n = 6) could not be detected by the conventional PCR and real-time PCR method. These results demonstrated that UNDP-PCR assay is greater sensitivity than the conventional PCR and real-time PCR methods. Meanwhile, the probes used in the assay were systematically selected and confirmed through serial experiments. These designs not only make the developed PCV2 UNDP-PCR assay obtain more wide detection breadth than antibody-based nanoparticle-amplification approaches, can detect different genotype and serotype PCV2 strains, but also make this assay more specific and economic.

## Conclusion

The nanoparticle DNA probe-based PCR assay developed in this study can reliably rule out false negative results from antibody-based assays, and is a DNA extraction free, high sensitive, specific, economic and rapid diagnosis method for preclinical PCV2 infection in field. This improved diagnostic method will probably affect measurements of PCV2 infection rate in field and offer a significant opportunity to identification of preclinical infection of PCV2 and evaluation of infection levels, and may provide a method for large-scale preclinical epidemiology investigation of PCV2, which will be helpful for prevention of PCV2 large-scale outbreaks.

## References

[1] Ellis J, Clark E, Haines D, West K, Krakowka S, et al (2004) Porcine circovirus-2 and concurrent infections in the field. Vet Microbiol 98: 159–163. 14741128 10.1016/j.vetmic.2003.10.008

[2] Opriessnig T, Meng XJ, Halbur PG (2007) Porcine Circovirus Type 2-Associated Disease: Update on Current Terminology, Clinical Manifestations, Pathogenesis, Diagnosis, and Intervention Strategies. Journal of Veterinary Diagnostic Investigation 19: 591–615. 17998548 10.1177/104063870701900601

[3] Finsterbusch T, Mankertz A (2009) Porcine circoviruses–small but powerful. Virus Res 143: 177–183. 19647885 10.1016/j.virusres.2009.02.009

[4] Segales J, Calsamiglia M, Olvera A, Sibila M, Badiella L, et al (2005) Quantification of porcine circovirus type 2 (PCV2) DNA in serum and tonsillar, nasal, tracheo-bronchial, urinary and faecal swabs of pigs with and without postweaning multisystemic wasting syndrome (PMWS). Vet Microbiol 111: 223–229. 16289542 10.1016/j.vetmic.2005.10.008

[5] Opriessnig T, McKeown NE, Harmon KL, Meng XJ, Halbur PG (2006) Porcine circovirus type 2 infection decreases the efficacy of a modified live porcine reproductive and respiratory syndrome virus vaccine. Clin Vaccine Immunol 13: 923–929. 16893993 10.1128/CVI.00074-06 PMC1539115

[6] Allan GM, Ellis JA (2000) Porcine circoviruses: a review. J Vet Diagn Invest 12: 3–14. 10690769 10.1177/104063870001200102

[7] Khayat R, Brunn N, Speir JA, Hardham JM, Ankenbauer RG, et al (2011) The 2.3-angstrom structure of porcine circovirus 2. J Virol 85: 7856–7862. 21632760 10.1128/JVI.00737-11 PMC3147897

[8] Mahe D, Blanchard P, Truong C, Arnauld C, Le Cann P, et al (2000) Differential recognition of ORF2 protein from type 1 and type 2 porcine circoviruses and identification of immunorelevant epitopes. J Gen Virol 81: 1815–1824. 10859388 10.1099/0022-1317-81-7-1815

[9] de Boisseson C, Beven V, Bigarre L, Thiery R, Rose N, et al (2004) Molecular characterization of Porcine circovirus type 2 isolates from post-weaning multisystemic wasting syndrome-affected and non-affected pigs. J Gen Virol 85: 293–304. 14769887 10.1099/vir.0.19536-0

[10] Kim JH, Lyoo YS (2002) Genetic characterization of porcine circovirus-2 field isolates from PMWS pigs. J Vet Sci 3: 31–39. 14614270

[11] Shuai J, Wei W, Li X, Chen N, Zhang Z, et al (2007) Genetic characterization of porcine circovirus type 2 (PCV2) from pigs in high-seroprevalence areas in southeastern China. Virus Genes 35: 619–627. 17851745 10.1007/s11262-007-0121-0

[12] Lefebvre DJ, Costers S, Van Doorsselaere J, Misinzo G, Delputte PL, et al (2008) Antigenic differences among porcine circovirus type 2 strains, as demonstrated by the use of monoclonal antibodies. J Gen Virol 89: 177–187. 18089741 10.1099/vir.0.83280-0

[13] Saha D, Huang L, Bussalleu E, Lefebvre DJ, Fort M, et al (2012) Antigenic subtyping and epitopes’ competition analysis of porcine circovirus type 2 using monoclonal antibodies. Vet Microbiol 157: 13–22. 22176764 10.1016/j.vetmic.2011.11.030

[14] Huang Y, Shao M, Xu X, Zhang X, Du Q, et al (2013) Evidence for different patterns of natural inter-genotype recombination between two PCV2 parental strains in the field. Virus Res 175: 78–86. 23545543 10.1016/j.virusres.2013.03.014

[15] Shao M, Lyu Y, Du Q, Shi L, Huang Y, et al (2013) Molecular epidemiological investigation on porcine circovirus type 2 in shaanxi province during 2010–2012. Journal of Northwest A&F university (nat scied) 41: 19–26.

[16] Wang ZS, Xu XG, Tong DW, Xing FS, Chen X, et al (2010) Cloning, sequence analysis and prokaryotic expression of ORF5 gene of PRRSV SX strain. Acta Agriculturae Boreali-occidentalis Sinica 19: 1–6.

[17] Shi L, Huang Y, Xu XG, Tong DW (2012) Sequence analysis of porcine parvovirus YL strain and prokaryotic expression of VP2. Acta Agriculturae Boreali-occidentalis Sinica 21: 6–12.

[18] Xu X, Guo H, Xiao C, Zha Y, Shi Z, et al (2008) In vitro inhibition of classical swine fever virus replication by siRNAs targeting Npro and NS5B genes. Antiviral Res 78: 188–193. 18262291 10.1016/j.antiviral.2007.12.012

[19] Xu XG, Chen GD, Huang Y, Ding L, Li ZC, et al (2012) Development of multiplex PCR for simultaneous detection of six swine DNA and RNA viruses. J Virol Methods 183: 69–74. 22575688 10.1016/j.jviromet.2012.03.034

[20] Sehgal D, Vijay IK (1994) A method for the high efficiency of water-soluble carbodiimide-mediated amidation. Anal Biochem 218: 87–91. 8053572 10.1006/abio.1994.1144

[21] Kim EY, Stanton J, Korber BT, Krebs K, Bogdan D, et al (2008) Detection of HIV-1 p24 Gag in plasma by a nanoparticle-based bio-barcode-amplification method. Nanomedicine (Lond) 3: 293–303. 18510425 10.2217/17435889.3.3.293 PMC2821699

[22] Nam JM, Thaxton CS, Mirkin CA (2003) Nanoparticle-based bio-bar codes for the ultrasensitive detection of proteins. Science 301: 1884–1886. 14512622 10.1126/science.1088755

[23] McIntosh KA, Tumber A, Harding JC, Krakowka S, Ellis JA, et al (2009) Development and validation of a SYBR green real-time PCR for the quantification of porcine circovirus type 2 in serum, buffy coat, feces, and multiple tissues. Vet Microbiol 133: 23–33. 18639395 10.1016/j.vetmic.2008.06.010

[24] Vlasakova M, Jackova A, Leskova V, Vilcek S (2012) Development of a Plexor real-time PCR assay for the detection of porcine circovirus type 2. J Virol Methods 179: 311–315. 22155430 10.1016/j.jviromet.2011.11.014

[25] Chen HT, Zhang J, Sun DH, Chu YF, Cai XP, et al (2008) Rapid detection of porcine circovirus type 2 by loop-mediated isothermal amplification. J Virol Methods 149: 264–268. 18355932 10.1016/j.jviromet.2008.01.023 PMC7112855

[26] Niemeyer CM, Adler M, Wacker R (2007) Detecting antigens by quantitative immuno-PCR. Nat Protoc 2: 1918–1930. 17703203 10.1038/nprot.2007.267

